# Coagulation biomarkers for ischemic stroke

**DOI:** 10.1016/j.rpth.2023.100160

**Published:** 2023-04-23

**Authors:** Aarazo Barakzie, A.J. Gerard Jansen, Hugo ten Cate, Moniek P.M. de Maat

**Affiliations:** 1 Department of Hematology, Erasmus MC, University Medical Center Rotterdam, Rotterdam, The Netherlands; 2 CARIM School for Cardiovascular Diseases, Maastricht University Medical Centre, The Netherlands; 3 Thrombosis Expertise Center and Department of Internal Medicine, Maastricht University Medical Centre, The Netherlands

**Keywords:** coagulation biomarkers, immunothrombosis, inflammation, ischemic stroke, prognosis, therapy

## Abstract

A State of the Art lecture titled “coagulation biomarkers for ischemic stroke” was presented at the International Society on Thrombosis and Haemostasis (ISTH) Congress in 2022. Ischemic stroke (IS) is a common disease with major morbidity and mortality. It is a challenge to determine which patients are at risk for IS or have poor clinical outcome after IS. An imbalance of coagulation markers may contribute to the progression and prognosis of IS. Therefore, we now discuss studies on the association of selected coagulation biomarkers from the hemostasis, inflammation, and immunothrombosis systems with the risk of IS, stroke severity at the acute phase, and clinical outcome after treatment. We report on coagulation biomarker–induced risk of IS, stroke severity, and outcomes following IS derived from prospective population studies, case-control studies, and acute-phase IS studies. We found indications that many coagulation and inflammation biomarkers are associated with IS, but it is early to conclude that any of these biomarkers can be applied in a therapeutic setting to predict patients at risk of IS, stroke severity at the acute phase, and clinical outcome after treatment. The strongest evidence for a role in IS was found for beta-thromboglobulin, von Willebrand factor, factor VIII, fibrinogen, thrombin-activatable fibrinolysis inhibitor, D-dimer, and neutrophil extracellular traps, and therefore, they are promising candidates. Further research and validation in large-size populations using well-defined study designs are warranted. Finally, we provide a selection of recent data relevant to this subject that was presented at the 2022 ISTH Congress.

## Essentials

• Ischemic stroke (IS) is associated with major morbidity and mortality.
• Imbalance in the coagulation system may play a role in the development and outcome of IS.
• We reviewed the association of coagulation markers with IS risk, stroke severity, and outcome.
• Finally, we summarized recent data on this topic presented at the 2022 ISTH Congress in London.

## 1 Introduction

Stroke is a common disease affecting 13.7 million people per year globally, with 5.5 × 10^6^ deaths annually [1,2]. It is estimated that 1 in 4 adults over the age of 25 years will experience a stroke over their lifetime and 1 in 3 will experience persistent complications [1].

Stroke is classified into 2 major types: ischemic stroke (IS) and hemorrhagic stroke. IS comprises 85% of all strokes. Causes of IS are diverse, including large cerebral artery occlusion by thrombosis, atherosclerosis, cardioembolism, or small vessel occlusion, and vary between races or ethnicities [[3], [4], [5]]. In this review, the term IS combines all different causes. Several studies showed that imbalance in the hemostasis, inflammation, and immunothrombosis systems may play a role in the development, progression, and clinical outcome of IS [[6], [7], [8], [9], [10]].

Prospective population-based cohort studies, with samples collected at a stable moment before or ±3 months after stroke onset, in which the main goal is to identify potential IS risk factors, showed that levels of most blood coagulation factors from primary and secondary hemostasis, fibrinolysis, inflammation, and immunothrombosis predict the risk of acute IS and its complications [[6], [7], [8]].

Also, studies conducted during the acute phase of IS (defined as IS onset and within 3 days thereafter) aimed to identify biomarkers that can predict stroke severity and clinical outcome (defined as neurologic deficit, functional disability, and mortality within 1 year after IS treatment). Less data are available from biomarkers at the acute phase of IS.

**Figure 1:**
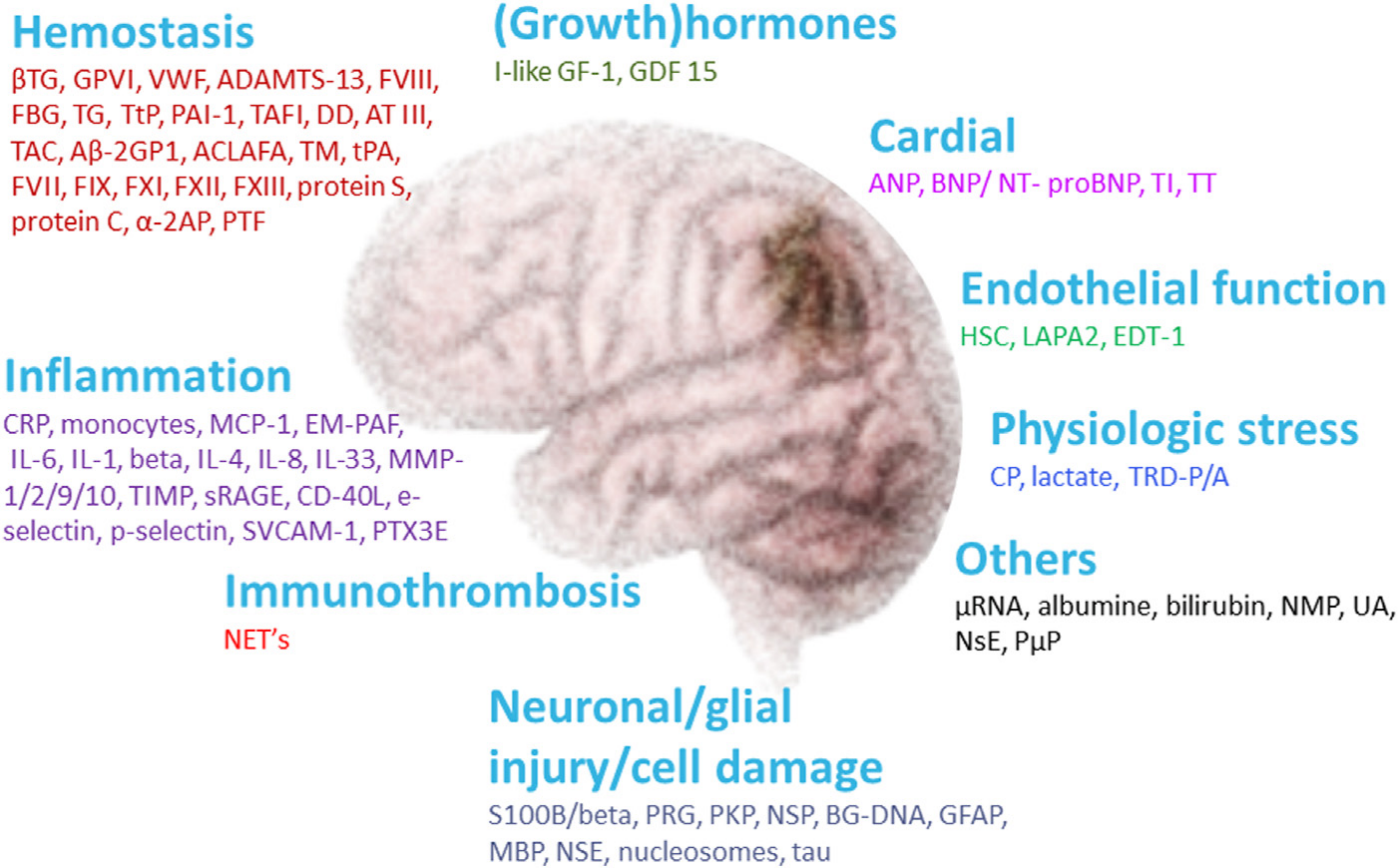
An overview of the potential biomarkers (also nonhemostasis-related) associated with an ischemic stroke that may predict the risk of stroke, stroke severity, and clinical outcome after treatment. In this review, we only discussed biomarkers of the hemostasis, inflammation, and immunothrombosis due to a close link between them. Hemostasis biomarkers: ßTG, GPVI, VWF, ADAMTS-13, FVIII, FBG, TG, TTP, PAI-1, TAFI, DD, AT III, TAT, Aß2GP1, AAFA, TM, tPA, FVII, FIX, FXI, FXII, FXIII, α-2AP, and PTF. Inflammation biomarkers: CRP, MCP-1, EM-PAF, IL-6, IL-1, beta IL-4, IL-8, IL-33, MP-1/2/9/10, TIMP, sRAGE, CD-40L, SVCAM-1, and PTX3e. Immunothrombosis biomarker: NETs. Neuronal/glial injury/cell damage biomarkers: S100B/ß, PRG, PKP, NSP, BG-DNA, GFAP, MBP, and NSE. (Growth) hormones: I-like GF-1 and GDF 15. Cardiac biomarkers: ANP, BNP/NT-proBNP, TI, and TT. Endothelial function biomarkers: HC, LAPA2, and EDT-1. Physiologic stress biomarkers: CP and TRD-P/A. Others: μRNA, NMP, UA, NSE, and E/Pμ. Abbreviations: AAFA, anticardiolipin antibodies fibrinopeptide A; ADAMTS-13, a disintegrin and metalloprotease with thrombospondin motif repeats 13; ANP, atrial natriuretic peptide; Aß2GP1, anti–beta-2 glycoprotein 1; AT III, antithrombine III; beta, IL-4=interleukin-6; BG-DNA, beta-globin DNA; BNP/NT-proBNP, brain natriuretic peptide; CD-40L, cluster of differentiation 40 ligand; CP, copeptin; CRP, C-reactive protein; DD, D-dimer; E/Pμ, endothelial/platelet microparticles; EDT-1, endothelin-1; EM-PAF, enhanced monocyte-platelet aggregate formation; FBG, fibrinogen; FIX, factor IX; FVII, factor VII; FVIII, factor VIII; FXI, factor XI; FXII, factor XII; FXIII, factor XIII; GDF 15, growth differentiation factor 15; GFAP, glial fibrillary acidic protein; GPVI, glycoprotein VI; HC, homocysteine; IL-1, interleukin-1; IL-33, interleukin-33; IL-6, interleukin-6; IL-8, interleukin-8; I-like GF-1, insulin-like growth factor-1; LAPA2, lipoprotein associated phospholipase A2; MBP, myelin basic protein; MCP-1, monocyte chemoattractant protein 1; MP-1/2/9/10, matrix metalloproteinase-1/2/9/10; NET, neutrophil extracellular trap; NMP, normetanephrine; NSE, neurone specific enolase; NSE, neurone specific enolase; NSP, neuroserpin; PAI-1, plasminogen activator inhibitor-1; PKP, proenkephalin; PRG, progranulin; PTF, prothrombin fragments; PTX3e, pentraxin-related protein expression; S100B/ß, S100 calcium-binding protein B; sRAGE; secretory receptor for advanced glycation end-products; ßTG, beta-thromboglobulin; SVCAM-1, selectin vascular cell adhesion molecule-1; TAFI, thrombin-activatable fibrinolysis inhibitor; TAT, thrombine-antitrombine complex; TG, thrombin generation; TI, troponin I; TIMP, tissue inhibitor of matrix metalloproteinase; TM, thrombomodulin; tPA, tissue-type plasminogen activator; TRD-P/A, thioredoxin paraoxonase/arylesterase; TT, troponin T; TTP, tissue-type plasminogen; UA, uric acid; VWF, von Willebrand factor; α-2AP, alpha-2 antiplasmin; μRNA, microRNA.

To predict individuals at risk of IS and to optimize management of patients with IS, several biomarkers have been explored, including markers for hemostasis, inflammation, and immunothrombosis (Figure 1), which are closely linked biologically. Coagulation and inflammation are essential parts of the defensive host response through endothelium, platelets, and proteases: 1) damaged endothelium expresses coagulation- and inflammation-related adhesive proteins, inductors, and receptors, 2) activated platelets release proteins with procoagulant and proinflammatory properties, and 3) blood-coagulation proteinases activate clotting and inflammation cells through their receptors. Immunothrombosis involves the formation of an intravascular thrombus through interaction between the innate immune system and hemostasis [11,12]. Only limited knowledge is available on the contribution of hemostasis, inflammation, and immunothrombosis factors to the risk of IS, stroke severity in the acute phase, and how these players relate to the response to initial treatment and clinically relevant outcomes.

Here, we present the association of selected blood biomarkers of hemostasis, inflammation, and immunothrombosis (for simplicity, referred to collectively as coagulation biomarkers) with the risk of IS, stroke severity in the acute phase, and therapy outcome.

## 2 Biomarkers of Primary Hemostasis in is

Disturbance in primary hemostasis may contribute to a prothrombotic state leading to IS (Table).

**Table:** Overview of coagulation biomarkers associated with risk of stroke and outcome.

Biomarker	References	Ischemic stroke risk in healthy population	Outcome after stroke	Outcome after tPA treatment
Primary hemostasis				
GPVI	[14,17]	-/↑	-/↑	-
β-TG	[[18], [19], [20], [21], [22]]	-/↑	-/↑	-/↑
Von Willebrand factor	[6,9,24,26,27]	↑	-/↑	↑
ADAMTS-13	[7,[27], [28], [29]]	↓	-/↓	-/↓
Secondary hemostasis				
Factor VIII	[9,[30], [31], [32], [33], [34], [35]]	↑	-/↑	↑
Fibrinogen	[[43], [44], [45], [46], [47], [48]]	↑	-/↑	-
Thrombin generation	[[37], [38], [39], [40]]	↓↑	-	↓
Fibrinolysis				
TPA	[[52], [53], [54]]	↑	-/↓	-
PAI-1	[[55], [56], [57], [58], [59], [60]]	↑	-/↑	-
TAFI	[[61], [62], [63], [64]]	↑	↑	↑
D-dimer	[[66], [67], [68], [69], [70]]	↑	↑	↑
Inflammation				
C-reactive protein	[[71], [72], [73],[75], [76], [77], [78]]	↑	↑	↓↑
TNF-α	[79,80]		↑	↑
Thrombus characteristics				
Mechanical, fibrin network		-	-	-
NETs	[10,12,88,89]	-	↑	-

β-TG, beta-thromboglobulin; ADAMTS-13, a disintegrin and metalloprotease with trompospondin motif repeats 13; eDNA, extracellular DNA; GPVI, glycoprotein VI; NET, neutrophil extracellular trap; PAI-1, plasminogen activator inhibitor-1; TAFI, thrombin-activatable fibrinolysis inhibitor; TNF-α, tumor necrosis factor alpha; TPA, tissue-type plasminogen activator.

↑ indicates an increase in biomarker; ↓ indicates a decrease in biomarker; and - indicates no changes in the levels of biomarker or insufficient data.

### 2.1 Platelets and their activation markers

Platelets are key players in thromboembolic diseases and are involved in the pathophysiological cascade resulting in IS. Increased levels of platelet activation markers have been shown in people with a risk of IS and in patients after IS [13].

#### 2.1.1 Glycoprotein VI

Platelet glycoprotein VI (pGPVI) is the central platelet membrane receptor for collagen binding, resulting in platelet activation and adhesion. pGPVI also improves thrombus growth and stabilization through binding to fibrin, which induces thrombin generation.

Limited studies have investigated the association of GPVI with risk for IS. One study demonstrated high pGPVI expression in patients at 3 months after IS; however, the expression of the dimeric form of pGPVI (pGPVI-dimer, increases GPVI avidity, causing further platelet activation) was even higher at 90 days after IS compared with that at admission [14].

Elevated pGPVI expression on admission was associated with poor clinical outcome at a 3-month follow-up in patients with IS, independent of usual laboratory markers C-reactive protein (CRP), blood glucose, and creatine kinase [15]. Further, elevated pGPVI and pGPVI-dimer expressions were associated with stroke severity at admission, but no association was found with functional outcome and death within 6 months [14]. Seyhan et al. [16] found no association between lower levels of pGPVI expression on admission and unfavorable 1-year outcome. Another study showed that increased pGPVI and pGPVI-dimer expressions on admission were associated with IS, whereas decreased plasma soluble glycoprotein VI levels were associated with acute cerebral ischemia; however, the association between soluble glycoprotein VI levels and pGPVI expression with clinical outcome was not reported [17].

#### 2.1.2 Beta-thromboglobulin

Beta-thromboglobulin (β-TG), a platelet activation marker, is normally present at very low levels in plasma. This marker is released from alpha-granules of platelets after platelet activation and activates platelet GPIIb/IIIa.

Limited studies have been conducted on the association of plasma β-TG levels with risk of IS. A study in a population with a history of stroke revealed a positive correlation between β-TG levels and time after IS (30 to 4000 days) in women; however, this correlation was negative in men [18]. Another study found that β-TG levels in patients who had experienced transient IS with high initial β-TG values within 24 hours from symptom onset were normal after a year [19].

Within the first 48 hours after IS, increased β-TG levels were associated with poor clinical outcome at 90 days following IS [20]. A similar study revealed that β-TG levels were increased at 48 hours from symptom onset in various subtypes of IS, including transient ischemic attacks and lacunar, atherothrombotic, and cardioembolic strokes, but there was no association between increased β-TG levels in the acute phase and infarct size in various subtypes of IS. Unfortunately, the relationship between levels and clinical outcome was not discussed in the study [21]. Another study showed that increased β-TG levels in the acute phase were associated with mortality at approximately 1 year following IS [22].

### 2.2 Von Willebrand factor

Von Willebrand factor (VWF) contributes to adhesion of platelets to the vascular wall following damage through binding to the platelet glycoprotein Ib receptor and forming bridges between platelets, thereby facilitating the earliest step of thrombus formation.

Several prospective population and case-control studies showed that plasma levels of VWF were associated with increased risk of IS incidence [6,23]. A prospective population study (Atherosclerosis Risk in Communities [ARIC]) including 14,700 participants showed that increased VWF levels were associated with IS incidence (n = 191) during 6 to 9 years of follow-up [6]. The Rotterdam Study, a large population-based cohort, included 6250 participants who were free from stroke at baseline. The risk of stroke increased with increasing VWF levels, after adjustment for age and sex [23].

A case-control study revealed that VWF levels were high in patients at 3 days after IS and documented the association between stroke severity, age, and sex and VWF levels [24]. Elevated VWF antigen levels immediately after thrombolysis and 24 hours after thrombolysis were independently associated with poor functional outcomes [9]. Another study at the acute moment of IS showed that VWF levels were increased in patients with increased neurologic deficit on admission and poor functional outcome at discharge [25]. An immunohistochemical study on emboli retrieved from patients with IS showed 2 types of platelet clusters in all emboli: VWF-positive platelets at the center and VWF-negative platelets surrounding them. High VWF-negative platelet content was correlated with response to thrombolysis, whereas high VWF-positive platelet and poor VWF patterns were correlated with increased number of attempts of mechanical clot removal [26].

### 2.3 A disintegrin and metalloprotease with the trompospondin motif repeat 13

A disintegrin and metalloprotease with the trompospondin motif repeat 13 (ADAMTS-13) enzyme cleaves large VWF multimers into smaller and less coagulant forms.

Limited studies are available on the association of ADAMT-13 with IS risk. In the prospective Rotterdam study, we showed that low ADAMTS-13 activity was significantly associated with risk of IS and predicted risk for IS [7]. A case-control study showed that low levels of ADAMTS-13 and high levels of VWF were associated with risk of IS in HIV–related IS [27].

Low ADAMTS-13 activity at admission was independently associated with unfavorable clinical outcomes in patients who underwent endovascular thrombectomy, while patients with unsuccessful reperfusion showed high levels of ADAMTS-13 at admission [28]. In contrast, another study on patients with IS showed no association between ADAMTS-13 levels at admission and unfavorable clinical outcomes 90 days after thrombolysis. However, ADAMTS-13 levels were lower at baseline in patients with unfavorable clinical outcomes at 90 days after thrombolysis than in patients with favorable clinical outcomes, but when the results were adjusted for age, history of atrial fibrillation, glycemia, baseline National Institute of Health stroke scale score, and Trial of Org 10172 in Acute Stroke Treatment classification, no difference in ADAMTS-13 levels was found [29].

## 3 Biomarkers Of Secondary Hemostasis in is

Secondary hemostasis consists of the extrinsic and intrinsic pathways (the contact pathway of coagulation). Both pathways result in common pathway activation and formation of the fibrin clot. Several of these coagulation factors have been linked to IS (Table).

### 3.1 Factor VIII

Factor VIII (FVIII) functions as a cofactor for factor IXa that cleaves inactive FX into its activated form (FXa). Increased FVIII levels are a common risk factor for venous thrombosis and may also be associated with risk of IS.

Several studies presented the association of FVIII with the risk of IS [[30], [31], [32], [33]]. The prospective population study ARIC that included 14,700 participants showed that increased FVIII levels were associated with IS incidence [6]. The Prospective Cohort with Incident Stroke Berlin (PROSCIS-B) study reported that high activity levels of FVIII were associated with IS recurrence or death during follow-up in the 3-year period after the first IS [30]. Similarly, a study in patients with worse outcomes after IS revealed that FVIII levels were increased during follow-up evaluation 8 to 10 months after IS [31]. In addition, the prospective cohort study REasons for Geographic and Racial Differences in Stroke (REGARDS) showed that elevated FVIII level was associated with increased risk of IS during a 4.5-year follow-up [32] and also revealed that increased FVIII level was associated with IS risk in patients with atrial fibrillation during a 5.2-year follow-up [33].

Various studies on IS have been performed on the association of FVIII levels in the acute phase with poor clinical outcome. Increased FVIII levels were associated with more severe neurologic impairments at presentation and higher neurologic deterioration during hospitalization [34]. Also, increased FVIII activity was independently associated with greater discharge disability, neurologic impairment, and recurrence of thromboses. Further, elevated FVIII activity immediately after and 24 hours after thrombolysis was independently associated with poor functional outcomes following IS [9]. No association was seen between FVIII levels at admission and prediction of vessel recanalization after thrombolysis [35]. At 24 hours after IS onset, increased FVIII levels were associated with poor clinical outcome at 90 days following IS [31].

### 3.2 Thrombin generation

Thrombin is an important enzyme in coagulation that promotes clot formation and also has several pleiotropic functions that link coagulation to atherosclerosis progression. Thrombin generation (TG) determines the overall balance of plasma pro- and anticoagulant factors. The main parameter of TG is endogenous thrombin potential, which refers to the net thrombin formation in a given plasma sample [36].

A case-control study in patients with IS and healthy controls indicated that TG started earlier, reached its peak faster, and finished earlier in patients with IS vs that in healthy controls at 1 to 3 months after the event, but there was no difference in endogenous thrombin potential between patients and controls [37]. Also, TG was associated with IS at a young age [37]. A prospective cohort study reported that higher TG at baseline was an independent risk factor for IS after 4 years of follow-up [38]. Another prospective cohort study indicated that TG at 1 month after IS was variable in patients with acute cardioembolic and noncardioembolic IS but was overall higher compared to that in healthy controls [39].

Only one study in the acute phase of IS reported on the association of TG with clinical outcome. This study found that low TG was an independent predictor of short- and long-term mortality in patients with IS postthrombolysis [40].

### 3.3 Fibrinogen

Fibrinogen is a glycoprotein generated in the liver that circulates in blood. This protein has a pivotal role in coagulation, although it has significant nonhemostatic functions as well, including a role in cellular and matrix interactions, inflammatory response, and wound healing. Fibrinogen is cleaved by the procoagulant enzyme thrombin into fibrin, the matrix of the clot [41]. Also, fibrinogen links platelets together via the platelet glycoprotein llb/llla, thereby forming a platelet plug [42]. Considerable interest has been shown in the relationship between fibrinogen and IS.

A meta-analysis of published data from 31 prospective studies showed a strong association of increased fibrinogen levels with IS risk, after adjustment for variables such as sex, smoking, blood pressure, and blood lipid levels in the regression model [43]. In a population study, ARIC, increased fibrinogen levels were associated with risk of IS after adjustment for multiple cardiovascular risk factors [6]. Additionally, increased levels of fibrinogen were independently associated with IS in young adults, whereas another study showed that increased fibrinogen levels were associated with the long-term cognitive outcome in young patients with IS at ≥3 months after the event [44,45].

Further, several studies showed an association of fibrinogen levels at the acute moment of IS with clinical outcomes [[46], [47], [48]]. High fibrinogen levels on admission were associated with poor functional outcome in patients with IS [46]. However, Vandelli et al. [47] reported that a reduction in fibrinogen levels increased the risk of intracerebral hemorrhage at 2 hours following thrombolysis in patients with IS. Potpara et al. [48] found that increased fibrinogen levels on admission were associated with poor clinical outcome in the acute phase of patients with IS.

## 4 Fibrinolysis in is

Fibrinolysis is an important process in the maintenance of the balance of the coagulation system. Abnormalities in the fibrinolytic system have been linked to increased risk of IS in a few studies (Table). In populations with coronary artery disease, fibrin formation and resistance to lysis are important determinants of outcome [49,50].

### 4.1 Tissue-type plasminogen activator

Tissue-type plasminogen activator (tPA), a serine protease, converts plasminogen into plasmin. Plasmin is a main fibrinolytic enzyme that degrades fibrin. Binding of tPA to plasminogen activator inhibitor-1 (PAI-1) abolishes its catalytic activity by forming an inactive PAI-1-tPA complex [51].

A prospective study demonstrated that high tPA levels in participants were associated with increased risk of IS in a 5-year follow-up period [52]. Another prospective study found that elevated tPA levels in plasma from patients who were prospectively followed over 5 years were independently associated with increased risk of IS [53].

On the other hand, reduced levels of tPA were shown to be associated with the diagnosis of IS, but this study did not document the relationship between tPA levels and clinical outcome [54]. A study in the acute phase of IS revealed that tPA levels were slightly, but not significantly, decreased in patients with poor clinical outcome [55].

### 4.2 Plasminogen activator inhibitor-1

PAI-1 is the primary inhibitor of tPA. Increased plasma PAI-1 levels have been linked to IS risk, severity on admission, and outcomes posttherapy [[55], [56], [57], [58], [59], [60]].

In a meta-analysis involving a total of 27 studies and 22,176 participants, elevated PAI-1 level was significantly associated with increased risk of IS in patients with arterial fibrillation [56]. A case-control study showed higher levels of PAI-1 in patients with IS than in healthy controls [57]. Another case-control study revealed that PAI-1 plasma levels measured 30 months prior to IS showed no direct association with the development of IS, but the tPA/PAI-1 complex was independently associated with the development of a first-ever stroke, especially hemorrhagic stroke [58].

A study in the acute phase of IS showed that increased PAI-1 levels on admission were associated with poor clinical outcome at 90 days [55]. A similar study on fibrinolytic profile at admission in patients with IS treated with tPA revealed that patients with good revascularization and favorable clinical outcomes exhibited significantly lower PAI-1 levels at admission as those with poor recanalization and unfavorable clinical outcome [59]. Interestingly, another study in the acute moment of IS demonstrated that increased levels of P-selectin (as indices of platelet activation) were associated with increased PAI-1 activity within 72 hours of IS onset, which may be correlated with decreased fibrinolysis in patients with IS [60].

### 4.3 Thrombin-activatable fibrinolysis inhibitor

Thrombin-activatable fibrinolysis inhibitor (TAFI) suppresses fibrinolysis by eliminating the plasminogen-binding site, which inhibits plasminogen activation. TAFI levels were elevated in men with symptomatic coronary artery disease and are risk factors for venous thrombosis [61], but limited studies are available on the level of TAFI in IS.

A study showed that increased functional TAFI levels, which were measured within 7 to 90 days after IS and indicate decreased fibrinolysis, are associated with an increased risk of IS [62]. A case-control study in individuals who were age- and sex-matched and had no history of vascular disease demonstrated that at 1 month after IS, higher functional TAFI levels in plasma increased the risk of recurrent IS [63].

Increased TAFI levels within 24 hours after IS were also related with worse neurologic outcome [64]. According to a study in the acute phase of IS, TAFI levels were higher in patients who underwent intravenous tPA therapy than in patients who did not undergo thrombolysis therapy. At 2 hours after thrombolysis, increased TAFI levels in plasma were associated with severe short-term outcome, whereas increased TAFI levels at 48 hours postthrombolysis were associated with unfavorable long-term outcome [65].

### 4.4 D-dimer

D-dimer is a degradation product of fibrin that is present in very low concentrations in the plasma of healthy individuals but is substantially increased in acute thrombotic or fibrinolytic events. The relationships of D-dimer with stroke risk, severity at the acute moment, and clinical outcomes have been investigated by several studies [[66], [67], [68], [69], [70]].

A meta-analysis including 22 prospective cohort and case-control studies showed that high D-dimer was associated with increased risk of IS [67]. Also, it was reported that high D-dimer on admission after onset of IS symptoms were correlated with increased risks of all-cause mortality, 5-day recurrence, and poor functional outcomes in these patients [67]. Large population-based studies, such as the ARIC and REGARDS studies, were included in this meta-analysis. Another study showed that elevated D-dimer at baseline was associated with a higher incidence of IS within a short time after admission of patients for acute heart failure [66]. A retrospective study demonstrated that D-dimer increased with age and was positively correlated with risk of IS in patients with nonvalvular atrial fibrillation. Additionally, in this patient group, baseline D-dimer did not predict IS, although D-dimer at IS onset (1.34 mg/L) was significantly (*P* <.001) increased compared with that at baseline (0.70 mg/L) [68].

A recent systematic review including 19 studies showed that elevated D-dimer was associated with poor functional outcome and higher mortality [69]. A prospective cohort study in the acute phase of IS found that at admission and 24 hours later, without any treatment, increased D-dimer correlated with the infarct lesion size and short-term clinical outcome [70].

## 5 Inflammation in is

### 5.1 C-reactive protein

CRP is an acute phase reactant and a sensitive marker of systemic inflammation, whose circulating concentrations rise in response to a wide range of acute and chronic inflammatory conditions such as bacterial or viral infections, necrosis, and tissue injury.

A meta-analysis of cohort studies with long follow-up (8 years) revealed that the risk of IS increased by nearly 70% in healthy individuals in the highest quartile of CRP concentrations compared to those in the lowest quartile [71]. A population study found that within 12 to 14 years of follow-up, high CRP levels predicted IS, independent of other cardiovascular risk factors [72]. A prospective nested case-control study demonstrated that CRP levels in patients followed-up for 12 years were positively associated with incident IS [73].

Two systematic reviews and meta-analysis including a total of 16 studies on IS reported that high CRP levels at the acute moment of IS were associated with poor clinical outcome, mortality, or risk of all-cause mortality at least 3 months of follow-up. [74,75]. A prospective study in the acute phase of IS showed that high levels of CRP within 24 hours after thrombolysis were not independently associated with poor clinical outcome [76]. Another study showed that the increased CRP level before thrombolysis correlated with mortality [77]. Napoli et al. [78] demonstrated that increased CRP level in the acute phase of IS at admission and discharge were associated with new vascular events or death at 1 year after the IS.

### 5.2 Tumor necrosis factor alpha

Tumor necrosis factor alpha (TNF-α) is an inflammatory cytokine released during the inflammatory state by monocytes, resulting in procoagulant changes in vascular endothelium by inducing the expression of tissue factor, reducing thrombomodulin (required for the anticoagulant effect of protein C), and increasing PAI-1.

No study reported on TNF-α as a risk factor for IS. A study at the acute phase of IS showed that increased TNF-α levels on admission were associated with lesion size and poor clinical outcome [79]. A similar study in the acute moment of IS showed that compared with baseline, TNF-a level significantly increased during the study until day 7, unrelated to lesion size and clinical outcome including neurologic impairment [80].

## 6 Thrombi Characteristics And Nets in is

### 6.1 Composition of stroke thrombi

The composition of stroke thrombi has been investigated in studies in which thrombi were retrieved by thrombectomy [[81], [82], [83]]. Histologic studies showed that a thrombus consists of fibrin, platelets, red blood cells (RBCs), white blood cells, extracellular DNA (a NETs marker), and VWF among other constituents. Two major thrombus areas are distinguished: 1) RBC-rich and fiber-poor area and 2) platelet-rich and fiber-rich area. Histologic staining indicated dense fibers throughout the platelet-rich area and a thin fiber network surrounding the RBC-rich area [84]. Thrombectomy-retrieved stroke thrombi also showed an RBC-rich area with dense fibrin mesh and aggregated platelets around it [85]. The retrieved thrombi were diverse in composition and structure that may also be dependent on early IS treatment [83,85]. Jolugbo et al. [82] reported that thrombus composition, size, location, and timing from IS onset were associated with IS imaging findings and clinical outcome. Future endovascular and thrombolytic treatments for stroke may be enhanced by developments in the detection or treatment of thrombi that consider clot heterogeneity.

### 6.2 Fibrin structure in stroke

Fibrin clot structure and function are altered in plasma clots of patients with IS [86]. Environmental and genetic factors, especially fibrinogen concentrations, affect fibrin clot properties. Fibrin has a tighter network with an increased diameter in patients with IS compared to controls [86].

Undas et al. [86] evaluated fibrin structure and function in patients with cryptogenic IS (strokes without a definite cause) 3 to 19 months after the event, and demonstrated that *ex vivo* plasma fibrin clots in patients had low permeability, faster fibrin polymerization, prolonged clot lysis time, higher maximum D-dimer released from clots, and a maximum rate of D-dimer release compared to controls. Cryptogenic IS was linked to altered fibrin structure and resistance to fibrinolysis.

Plasma fibrin clots at admission were denser than fibrin clots formed in the plasma of patients collected at 24 hours following thrombolysis. Variables involved in fibrin clot properties, including lower maximum absorbance of fibrin gels, shorter clot lysis time, and increased D-dimer at baseline, were associated with favorable functional outcome (modified Rankin Scale score 0-2) at 3 months after adjustment for age and fibrinogen, whereas denser fibrin formation indicated poor lysability and outcome [87].

Neutrophil extracellular traps (NETs) are networks of extracellular DNA decorated with histones and granular proteins such as myeloperoxidase and neutrophil elastase released, during the acute phase of a disease, by activated neutrophils. NETs assist immunothrombosis via several pathways, including direct activation of FXII, platelet recruitment, triggering of platelet activation by H3 and H4 present in NETs, promotion of the activation of the extrinsic pathway, and inactivation of natural anticoagulants. A role of NETs in thrombus formation in small vessel IS has been described, which was not seen in the other subtypes of IS [12].

Histologic studies indicated that NETs are present in ischemic thrombi, particularly in the outer layer of thrombi. NETs are more often seen within platelet-rich areas and at the boundary between platelet-rich and red blood cell–rich areas of thrombi [84]. In thrombi retrieved from patients with IS, the NET content impaired tPA-induced thrombolysis. NETs targeting with DNase 1 promoted *ex vivo* lysis of IS thrombi [88]. The abundance of NETs in thrombi retrieved from patients with IS was associated with poor clinical outcome [10]. Also, elevated NET levels at admission were associated with stroke severity and mortality in patients with IS [89].

## 7 Isth Congress Report

Several studies aiming to identify the potential biomarkers for outcome after IS were presented at the International Society on Thrombosis and Haemostasis (ISTH) 2022 in London.

### 7.1 α2-plasmin inhibitor in IS

α2-plasmin inhibitor (α2-PI) is a key regulator of fibrinolysis. Alteration in α2-PI may contribute to vessel occlusion, leading to IS, as well as recanalization failure or bleeding problems in patients with IS. α2-PI has not been much studied as a risk factor for IS or as a predictor of thrombolysis outcomes. During the ISTH Congress 2022, Székely et al. [90] presented a large prospective study in which α2-PI dropped immediately after thrombolysis and increased to subnormal levels 24 hours after thrombolysis. Low α2-PI levels at admission were associated with stroke severity and unfavorable long-term outcomes.

### 7.2 PAI-1 4G/5G in IS

The 675 4G/5G polymorphisms in the *PAI-1* gene affect PAI-1 levels. The 5G allele possesses a transcription repressor-binding site that partially overlaps the activator-binding site. Therefore, individuals with the genotype 4G/4G have higher levels of PAI-1 than those with the genotype 5G/5G PAI-1. A recent meta-analysis suggests that the PAI-1 4G/5G polymorphism may serve as a genetic biomarker for IS stroke risk [91]. Limited studies are reported on the association of PAI-1 4G/5G polymorphism with outcome after IS. During the ISTH 2022 Congress, Szegedi et al. [92] presented that PAI-1 levels and 4G/5G polymorphism were not associated with long-term outcomes, but PAI-1 4G/5G polymorphism was associated with intracranial hemorrhage postlysis.

## 8 Conclusion and Future Directions

Based on the publications discussed in this manuscript, it is clear that many coagulation and inflammation biomarkers are associated with IS, but it is premature to conclude that coagulation biomarkers can be used to identify which patients are at risk of IS, stroke severity, or a poor clinical outcome. β-TG, VWF, FVIII, fibrinogen, D-dimer, TAFI, and NETs were associated with risk of IS, stroke severity at the acute moment, and clinical outcome after treatment, and for that matter, they may be the most interesting candidates. The presence of the coagulation factors in thrombi retrieved from patients with IS and their association with thrombolysis and the number of attempts of mechanical clot removal also adds to the importance of the coagulation factors in the progression of IS and its treatment. However, due to the inhomogeneity in the studies, including clinical and methodological variability, treatment types, period of clinical outcome evaluation, the timing of blood sampling, and nonspecification of the exact interval between the onset of symptoms and discharge, it is difficult to compare the findings across studies and make a firm conclusion. In addition, we combined the literature on different subtypes of IS in this review due to the limited data available on all subtypes of IS on biomarkers and outcome. Further research is needed to determine biomarkers for each subtype of IS.

**Figure 2:**
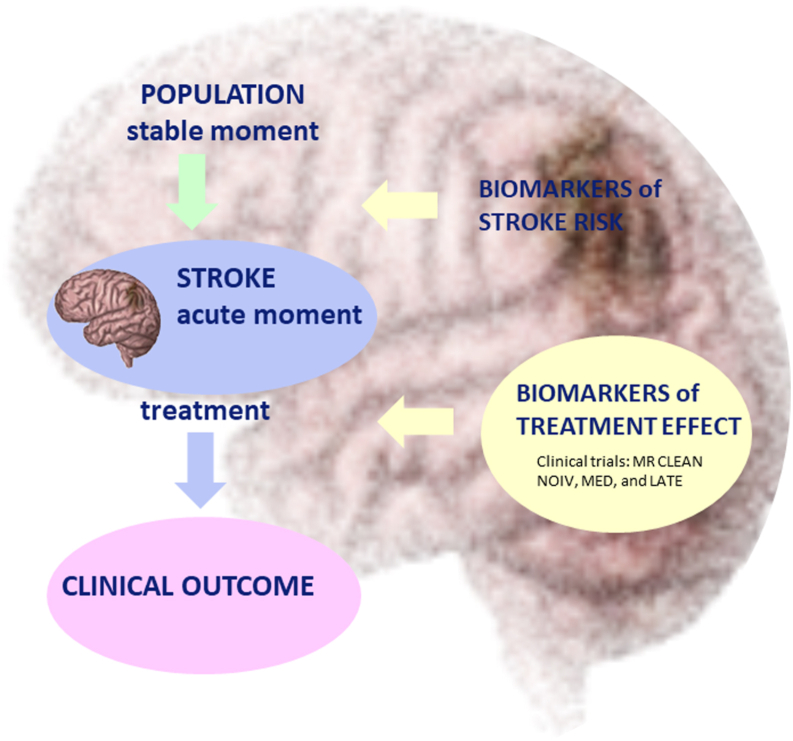
Future directions. Studies at the stable moment will help identify biomarkers that are associated with stroke risk [[6], [7], [8]], whereas studies at the acute moment of stroke will help identify biomarkers that can predict treatment effect and thereby clinical outcome, including clinical trials MR CLEAN NOIV, MR CLEAN MED, and MR CLEAN LATE [[95], [96], [97]].

In the future, large-size patient studies are required to identify which biomarkers are involved in the prediction of IS progression (Figure 2). At the moment, several reperfusion options are available, including thrombolytic therapy (recombinant tissue-tPA [rt-tPA]) and endovascular thrombectomy. Thrombolytic treatment given within 3 to 4 hours of the onset of symptoms is safe and effective in selected patients with IS. Only 30% to 40% of selected patients can benefit from thrombolysis [93]. Endovascular thrombectomy, ie, mechanical retrieval of the thrombus via catheter angiography, has been shown by MR CLEAN and other studies to be able to improve outcomes in patients with a large vessel occlusion (LVO), when performed within 6 hours from symptom onset and in selected patients based on clinical and imaging parameters up to 24 hours after IS onset [94]. Despite this great improvement, the effect of reperfusion and therefore the clinical outcome cannot be predicted after IS. Reperfusion by any method is also associated with ischemia/reperfusion brain injury. Only limited knowledge is available on the contribution of coagulation factors in the acute phase of IS, response to treatment, and clinical outcomes. Additional studies with sufficient power, clearly defined inclusion and exclusion criteria, serial biomarker measurements at various time points during the acute phase, and adequate statistical adjustments for potential confounders will be required to identify which coagulation biomarkers can be used in the clinical setting. Currently, randomized clinical trials, including MR CLEAN-NOIV, MED, and LATE, which collected blood samples in the acute phase before, immediately after, and 24 hours after reperfusion therapy, as well as during follow-up (3 months), are examining the efficacy and safety of various acute IS treatments in 2500 patients [[95], [96], [97]]. The MR CLEAN-NOIV trial investigates the added benefit of intravenous alteplase prior to intra-arterial thrombectomy in patients with IS and an intracranial LVO of the anterior circulation (ILVO-AC), within 4.5 hours after symptom onset. The MR CLEAN-MED trial studies the effect of periprocedural medication—heparin, antiplatelet agents, both, or neither—in patients with IS and ILVO-AC admitted within 6 hours from symptom onset. The MR CLEAN-LATE trial compares the efficacy of endovascular treatment to the best medical care in patients with ILVO-AC–induced acute IS who arrive between 6 and 24 hours after onset. These different studies will help us gain a better understanding of the timing of blood biomarker assessment in relation to clinical outcomes in patients with IS (Figure 2).
